# Clinical and functional outcomes at 7-year follow-up of children presenting putative antecedents of schizophrenia at age 9-12 years

**DOI:** 10.1038/s41537-024-00507-8

**Published:** 2024-09-30

**Authors:** Alexis E. Cullen, Ruth E. Roberts, Helen L. Fisher, Kristin R. Laurens

**Affiliations:** 1 Department of Psychosis Studies, Institute of Psychiatry, Psychology & Neuroscience, King’s College London, London, UK; 2 Division of Insurance Medicine, Department of Clinical Neuroscience, Karolinska Institutet, Stockholm, Sweden; 3 Education & Training Division, Anna Freud, London, UK; 4 Research Department of Clinical, Educational & Health Psychology, University College London, London, UK; 5 Institute of Psychiatry, Psychology & Neuroscience, Social, Genetic & Developmental Psychiatry Centre, King’s College London, London, UK; 6 ESRC Centre for Society and Mental Health, King’s College London, London, UK; 7 School of Psychology and Counselling, Queensland University of Technology (QUT), Brisbane, QLD Australia

**Keywords:** Schizophrenia, Psychosis

## Abstract

Identification of youth presenting early risk factors for psychosis may facilitate preventive intervention. Through school-based screening, we recruited 112 children aged 9–12 years who presented multiple putative antecedents of schizophrenia (ASz), a family history of schizophrenia (FHx), or neither of these risk factors (typically-developing; TD). Clinical and functional outcomes were assessed at age 17–21 years (*N* = 93). Compared to the TD group, the ASz group had higher total Prodromal Questionnaire (PQ) scores (*β* = 10.59, 95% CI = 3.76, 17.42) and total psychopathology scores (*β* = 6.13, 95% CI: 1.03, 11.23), were more likely to score above-threshold on the PQ positive symptoms scale (OR = 4.00, 95% CI = 1.08, 14.83), and had lower scores on the Social and Occupational Functioning Scale (*β* = –9.43, 95% CI = –15.08, –3.77) at follow-up. The FHx and TD groups did not differ on any outcome. Findings suggest that population screening for putative antecedents of schizophrenia may identify children who would benefit from preventative intervention.

## Introduction

Psychotic disorders affect ~1% of the population during their lifetime^1^. Given their early age of onset^2^, and potentially chronic course if sub-optimally treated^3^, there has been significant research and clinical investment in identifying individuals during the early stages of illness^4^. Several strategies have been developed to identify young people with increased risk of developing psychotic disorders. The most widely adopted of these has been the clinical high-risk (CHR) approach^5,6^, an indicated prevention strategy targeting help-seeking individuals (typically aged 12–35 years^7^) who present with attenuated psychotic symptoms (APS) (i.e. positive psychotic symptoms that are not sufficiently severe and/or frequent to meet criteria for full-threshold psychotic disorder but are nonetheless distressing), or, less-commonly, brief and limited intermittent psychotic symptoms, or trait liability (schizotypal personality disorder or a family history of psychosis) plus functional decline. This approach has been highly influential, with specialist CHR treatment services now implemented throughout the world^8^. From a prognostic perspective, CHR status is the most robust risk factor for psychotic disorders^9^, with a recent meta-analysis of 130 studies (mean participant age: 20.3 years) indicating that around a quarter of CHR individuals transition to psychosis within 2–3 years^10^. However, a meta-analysis of data from youth aged ≤18 years observed markedly lower transition rates (12.1% at 2 years) and found no evidence to suggest that CHR status was associated with increased risk after accounting for risk-enrichment strategies^11^. One further concern is the limited evidence that transition risk can be reduced among individuals at CHR (irrespective of age) with specific interventions^10,12^. Moreover, this population is characterised by clinical comorbidity, functional impairment, and cognitive deficits^6,13^; fewer than half of those who do not transition achieve remission from the CHR state^14^, and even those who do remit continue to show functional impairments^15^.

The high levels of disability and impairment associated with the CHR state, irrespective of transition risk, suggest that it may be beneficial to supplement this indicated prevention strategy with a selective prevention approach targeting population subgroups with above-average risk. Such approaches require widescale screening efforts to identify young people with psychosis risk factors before they seek clinical care^5^. Our group has developed such an approach, which uses a school-based screening method to identify children aged 9–12 years who present multiple putative antecedents of schizophrenia (ASz). These antecedents are thought to represent early manifestations of disease; as such, they can be distinguished from ‘risk factors’, which include more passive and/or environmental markers of risk, such as experiences of trauma/adversity or cannabis use, which may pose ethical dilemma when assessed in children (in the United Kingdom, researchers have a duty of disclosure that requires them to act on information received regarding a risk of harm to the participant or others, if aged <18 years. For this reason, there would be ethical dilemma associated with selecting children to participate in research on the basis of risk factors that require the research team to act on these disclosures). To facilitate screening of community samples, we included factors that were amenable to questionnaire assessment and did not require professional assessment (e.g. IQ). Based on longitudinal studies available at commencement of our study, we selected three putative ASz: (i) speech and/or motor delays, (ii) social, emotional and/or behavioural problems, and (iii) psychotic-like experiences (PLEs)^16–19^.

PLEs, which can be assessed via self-report or interview methods, are typically defined as alterations in perception and reality that occur at the subclinical level^20^. These experiences are qualitatively different to the APS that characterise individuals at CHR^21^ in that they are less commonly distressing and/or intrusive^19^. PLEs were included as one of the three putative ASz based on evidence that interview-reported hallucinations and delusions at age 11 years were associated with a 16-fold increase in the risk of schizophreniform disorder at age 26^22^. Since then, literature on PLEs has burgeoned, with meta-analyses indicating that PLEs are associated with a fourfold increase in the risk of developing psychotic disorders and a threefold increase in risk for any mental disorder^23^. However, there are concerns that, given the high prevalence of PLEs in young children and adolescents (median prevalence rates of 17% vs. 7.5%, respectively^24^), PLEs in youth may not be as pathognomonic as those experienced in adulthood^25^ with the majority of PLEs experienced in this age group being transitory and benign^26^. Indeed, a recent study found that self-reported PLEs had poorer ability to predict CHR/psychosis status at interview in early adolescence (mean age 13 years) than in mid- and older adolescence (mean age 15 and 18 years, respectively)^27^. Whilst these more recent findings suggest that PLEs alone might not be suitable for selective preventative approaches, we reasoned that the presence of multiple putative ASz in childhood would provide a more sensitive and specific means of identifying those at high risk for schizophrenia spectrum disorders.

The present study utilises data from the London Child Health and Development Study (CHADS)^16^, a longitudinal investigation of children who presented the triad of putative ASz at age 9–12 years. Children with a family history of schizophrenia/schizoaffective disorder (FHx), a well-established high-risk population, were recruited as a comparison group to enable us to differentiate risk markers that may be primarily genetically mediated (and may be shared by non-symptomatic relatives) from others present only in individuals displaying the ASz triad. These two ‘high-risk’ groups were compared to typically-developing (TD) children who presented none of the putative antecedents nor a family history at recruitment. The aim of the present study was to describe the clinical and functional characteristics of the putative ASz group at age 17–21 years, this age range represents a key developmental stage that captures the peak incidence for psychosis and other major psychiatric disorders^2^. We hypothesised that both ASz and FHx groups would show poorer clinical and functional outcomes at follow-up relative to their TD peers. As we have previously observed (using data from earlier follow-up assessments) that ASz and FHx groups are characterised by cognitive impairments^28–30^ and increased sensitivity to daily stressors^31^ relative to TD children, we explored whether any group differences in clinical and functional outcomes might be explained by these factors. The latter was particularly relevant given that in a large, partially overlapping sample (including youth who did not meet ASz, FHx, or TD criteria) daily stressors at age 11–14 years were independently associated with the emergence of APS at age 17–21 years^32^.

## Results

### Participation at follow-up

At wave 1, 112 children aged 9–12 years who fulfilled ASz (*n* = 41), FHx (*n* = 26), or TD (*n* = 45) criteria were recruited to the longitudinal study (this includes 6 children who also had a 1st or 2nd degree relative with schizophrenia/schizoaffective disorder, 4 of whom participated in the follow-up assessment at age 17–21 years and are included in the present analyses; these children were eligible for the FHx group but are included in the ASz group for the main analyses). Of these, 93 (83%) completed the wave 4 assessment at age 17–21 years (mean [±SD] lapse-of-time between screening and follow-up assessment: 7.21 [±1.10] years); follow-up rates for ASz, FHx, and TD groups were 80%, 77%, and 89%, respectively. The remaining 19 individuals either declined participation (*n* = 6), could not be scheduled (*n* = 4) or contacted (*n* = 4), or were found at interim assessment phases to meet study exclusion criteria (*n* = 5). Individuals who participated in the current assessment phase did not differ from those lost to follow-up (for any reason) on group status at recruitment, age at screening, sex, ethnicity, or daily stressor score at wave 2 (*P* > 0.05 for all, see Supplementary Table 1) but did differ on caregiver occupation (*P* = 0.009) and IQ at wave 1 (*P* = 0.004). Specifically, the caregivers of participants who were lost to follow-up were more likely to be employed in routine and manual occupations and less likely to have managerial/professional occupations than the caregivers of those who were followed up at wave 4, and participants who were lost to follow-up had lower wave 1 IQ scores than those who completed wave 4 assessments.

### Group differences in sociodemographic variables

Sociodemographic characteristics of the 93 participants who completed the wave 4 assessment are provided in Table 1 by risk group. ASz, FHx, and TD groups differed on ethnicity, caregiver occupation at wave 1, sex, IQ at wave 1, and daily stressor score at wave 2 (though sex differences did not reach statistical significance, *P* = 0.051). No significant group differences in age at screening, age at wave 4, or lapse-of-time between screening and wave 4 were observed (*P* > 0.05 for all).

**Table 1:** Sociodemographic characteristics of participants assessed at the wave 4 assessment (age 17-21 years).

		Sample assessed at wave 4 (*N* = 93)						
Characteristics	Wave	ASz (*n* = 33)		FHx (*n* = 20)		TD (*n* = 40)		Statistical test
Age at screening (years), Med. (IQR)	SC	10.35	(1.59)	10.47	(0.94)	10.28	(1.28)	*KW* = 0.20, *P* = 0.906
Age at follow-up (years), Med. (IQR)	4	17.43	(1.00)	17.27	(0.68)	17.22	(0.62)	*KW* = 5.75, *P* = 0.057
Time lapse (years), mean (SD)	–	7.29	(1.23)	7.01	(1.16)	7.24	(0.96)	*KW* = 1.32, *P* = 0.518
Sex (male), *n* (%)	1	21	(63.6)	6	(30.0)	18	(45.0)	*χ*^*2*^ = 5.96, *P* = 0.051
Ethnicity, *n* (%)	1							
White		15	(45.5)	4	(20.0)	30	(75.0)	*FE*, *P* = 0.001
Black		11	(33.3)	8	(40.0)	5	(12.5)	
Other		7	(21.2)	8	(40.0)	5	(12.5)	
Caregiver occupation, *n* (%)	1^a^							
Managerial/professional		15	(46.9)	13	(65.0)	35	(87.5)	*FE*, *P* = 0.002
Intermediate		10	(31.3)	3	(15.0)	4	(10.0)	
Routine and manual		7	(21.9)	4	(20.0)	1	(2.5)	
Full-scale IQ, mean (SD)	1^b^	102.2	(12.9)	102.5	(16.4)	115.0	(11.9)	*KW* = 17.70, *P* < 0.001
Daily stressor PC score, mean (SD)	2	0.72	(1.19)	–0.23	(1.04)	–0.33	(1.22)	*KW* = 14.14, *P* < 0.001

*ASz* antecedents of schizophrenia, *FHx* family history of schizophrenia/schizoaffective disorder, *TD* typically developing, *SC* screening phase, *Med* median, *IQR* interquartile range, *SD* standard deviation, *KW* Kruskal–Wallis test, *FE* Fisher’s exact test, *PC* principal component.

^a^Collected at wave 2 (*n* = 6) or 3 (*n* = 4) due to missing data at wave 1.

^b^Collected at wave 2 (*n* = 3) and wave 3 (*n* = 2) due to missing data at wave 1. Missing data, caregiver occupation (*n* = 1); daily stressor PC score (*n* = 6).

### Clinical and functional outcomes at follow-up

#### Descriptive statistics

Descriptive statistics for all outcome variables are provided in Table 2. Median total Prodromal Questionnaire (PQ) scores were highest in the FHx group, intermediate in the ASz group, and lowest in the TD group. Around one-third of individuals in the ASz (33.3%) and FHx (35.0%) groups scored ≥8 on the PQ positive scale compared to 15.0% of the TD group. Applying the more stringent cut-off (≥18 on the positive scale), 21.2%, 0.0%, and 2.5% of ASz, FHx, and TD individuals, respectively, scored above threshold. Mean total youth self-report (YSR) T-scores were highest in the ASz group but similar in the FHx and TD groups. In terms of functioning, most individuals were currently engaged in full- or part-time education, although the proportion was somewhat lower in the ASz group (81.8%) compared to the FHx (95.0%) and TD groups (97.5%); a third of participants in all groups were employed on a full- or part-time basis. In the total sample, 5 individuals (5.4%) were not engaged in education or employment, which was somewhat more common in the ASz group (9.1%). Social and Occupational Functioning Scale (SOFAS) scores were lowest in the ASz group (median: 73, corresponding to ‘slight impairment’) and highest in the TD group (median: 85, corresponding to ‘good functioning’), with individuals in the FHx group exhibiting intermediate scores (median: 80, corresponding to ‘good functioning’). Within the total sample, seven individuals met Comprehensive Assessment of At-Risk Mental States (CAARMS) CHR criteria (6 due to APS, 1 due to trait vulnerability) and one participant (within the ASz group) reached CAARMS psychosis threshold. Across the ASz, FHx, and TD groups, respectively, 9.1%, 10.0%, and 7.5%, met CHR or FEP (first-episode psychosis) criteria as determined using the CAARMS.

**Table 2:** Descriptive statistics for clinical and functional outcomes at 7-year follow-up (wave 4).

	ASz (*n* = 33)		FHx (*n* = 20)		TD (*n* = 40)	
PQ total score, Med. (IQR)	16.0	(25.0)	18.5	(17.5)	8.5	(9.0)
PQ cut-off ≥8 positive scale, *n* (%)	11	(33.3)	7	(35.0)	6	(15.0)
PQ cut-off ≥18 positive scale, *n* (%)	7	(21.2)	0	(0.0)	1	(2.5)
YSR Total T-score, mean (SD)	53.3	(9.6)	47.0	(10.4)	47.2	(9.2)
Currently in education, *n* (%)	27	(81.8)	19	(95.0)	39	(97.5)
Currently in employment, *n* (%)	13	(39.4)	7	(35.0)	13	(32.5)
Not in education or employment, *n* (%)	3	(9.1)	1	(5.0)	1	(2.5)
SOFAS score, Med. (IQR)	73.0	(20.0)	80.0	(15.0)	85.0	(10.0)
CAARMS CHR or psychosis criteria met, *n* (%)	3	(9.1)	2	(10.0)	3	(7.5)

*ASz* antecedents of schizophrenia, *FHx* family history of schizophrenia/schizoaffective disorder, *TD* typically developing, *PQ* prodromal questionnaire, *Med* median, *IQR* interquartile range, *YSR* youth self-report, *SOFAS* social and occupational functioning assessment scale, *CAARMS* comprehensive assessment of at-risk mental states, *CHR* clinical high-risk. Missing data, YSR Total T-score (*n* = 8).

#### Multivariable models

**Fig. 1:**
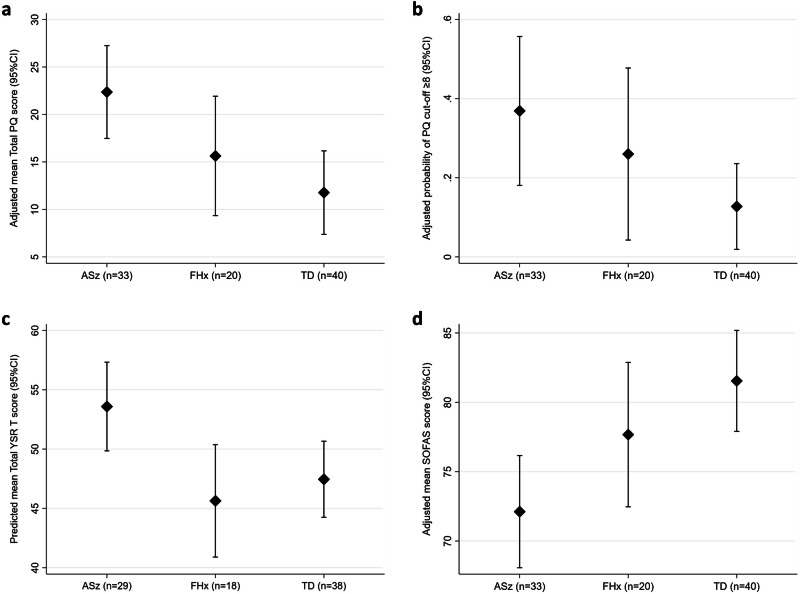
Clinical and functional outcomes at wave 4 (age 17–21 years) by risk status at wave 1 (age 9–12 years). Plots show adjusted means (total PQ, YSR, and SOFAS scores, panels **a**, **c**, and **d**) and adjusted predicted probabilities (PQ threshold, panel **b**) with 95% CIs, derived from linear and logistic regression models examining associations between group status at wave 1 and outcomes at follow-up. All models are adjusted for sex, ethnicity, and caregiver occupation at baseline. ASz individuals presenting all three putative antecedents of schizophrenia at wave 1, FHx individuals with a family history of schizophrenia/schizoaffective disorder, TD individuals recruited as typically-developing control group, PQ prodromal questionnaire, YSR youth self-report, SOFAS social and occupational functioning scale, CI confidence interval.

Multivariable regression models were performed to examine associations between risk group status and total PQ scores, total YSR T-scores, SOFAS scores, and PQ threshold scores on the positive scale (≥8 above) at wave 4 (Table 2). Regression analyses were not performed on CAARMS outcomes due to the small number of individuals meeting CHR/psychosis criteria. After adjusting for sex, ethnicity, and caregiver occupation (model 1), individuals meeting ASz criteria at recruitment had higher total PQ scores (*β* = 10.59, 95% CI: 3.76, 17.42) and total YSR T-scores (*β* = 6.13, 95% CI: 1.03, 11.23), were more likely to score above threshold on the PQ positive scale (OR = 4.00, 95% CI: 1.08, 14.83), and had lower SOFAS scores (*β* = –9.43, 95% CI: –15.08, –3.77) at wave 4 when compared to the TD group. The ASz group also showed significantly higher total YSR T-scores when compared to the FHx group (*β* = 7.95, 95% CI: 1.81, 14.08) but did not differ on other measures. In contrast, no significant differences on any of the four outcome measures were observed between the FHx and TD groups. Figure 1 shows the adjusted means (total PQ, total YSR T-scores, and SOFAS scores) and adjusted predicted probabilities (PQ threshold) derived from model 1, respectively, by risk group status. This figure shows that, after accounting for sex, ethnicity, and caregiver occupation at wave 1, the ASz group were predicted to show the poorest outcomes among the three groups on all measures.

#### Role of IQ and daily stressors

We additionally explored whether risk group status remained independently associated with outcomes after additionally adjusting for IQ at wave 1 and daily stressor score at wave 2 (Table 3). All significant group differences observed between the ASz and TD groups and between the ASz and FHx groups in model 1 remained after adjustment for IQ. Here, coefficients were largely unchanged except for the difference in SOFAS scores between the ASz and TD groups, which was slightly attenuated (*β* = –7.10, 95% CI: –12.54, –1.66). In contrast, after adjustment for daily stressor scores (model 3), differences between the ASz and TD groups across all four outcome measures were attenuated, such that significant differences were observed for total PQ scores only (*β* = 8.21, 95% CI: 0.98, 15.45). The significant differences observed between ASz and FHx groups on total YSR T-scores also somewhat attenuated (*β* = 6.49, 95% CI: 0.21, 12.77).

**Table 3:** Association between risk status at recruitment and clinical and functional outcomes at 7-year follow-up (wave 4) in multivariable regression models adjusted for sex, ethnicity, and caregiver occupation at wave 1 (model 1) with additional adjustment for full-scale IQ at wave 1 (model 2) and daily stressors at wave 2 (model 3).

	Total PQ score			PQ cut-off			Total YSR T-score			SOFAS score		
Pairwise comparisons	*β*	(95% CI)	*P*	OR	(95% CI)	*p*	*β*	(95% CI)	*P*	*β*	(95% CI)	*P*
Model 1												
ASz vs. TD (ref)	**10.59**	**(3.76, 17.42)**	**0.003**	**4.00**	**(1.08, 14.83)**	**0.038**	**6.13**	**(1.03, 11.23)**	**0.019**	**–9.43**	**(–15.08, –3.77)**	**0.001**
ASz vs. FHx (ref)	6.73	(–1.35, 14.80)	0.101	1.66	(0.40, 6.96)	0.486	**7.95**	**(1.81, 14.08)**	**0.012**	–5.55	(–12.24, 1.13)	0.102
FHx vs. TD (ref)	3.86	(–4.08, 11.81)	0.336	2.41	(0.57, 10.07)	0.229	–1.82	(–7.73, 4.09)	0.542	–3.87	(–10.45, 2.70)	0.245
Model 2												
ASz vs. TD (ref)	**10.58**	**(3.51, 17.65)**	**0.004**	**3.98**	**(1.05, 15.17)**	**0.043**	**6.75**	**(1.53, 11.97)**	**0.012**	**–7.10**	**(–12.54, –1.66)**	**0.011**
ASz vs. FHx (ref)	6.73	(–1.40, 14.85)	0.104	1.66	(0.40, 6.96)	0.486	**8.13**	**(1.99, 14.27)**	**0.010**	–5.29	(–11.55, 0.97)	0.096
FHx vs. TD (ref)	3.85	(–4.28, 11.98)	0.349	2.40	(0.56, 10.22)	0.238	–1.38	(–7.34, 4.58)	0.646	–1.81	(–8.07, 4.45)	0.567
Model 3												
ASz vs. TD (ref)	**8.21**	**(0.98, 15.45)**	**0.027**	3.11	(0.70, 13.85)	0.137	4.62	(–0.63, 9.87)	0.083	–6.25	(–12.55, 0.04)	0.051
ASz vs. FHx (ref)	4.80	(–3.60, 13.21)	0.259	1.31	(0.24, 7.13)	0.757	**6.49**	**(0.21, 12.77)**	**0.043**	–2.27	(–9.58, 5.04)	0.538
FHx vs. TD (ref)	3.41	(–4.31, 11.13)	0.382	2.38	(0.50, 11.37)	0.277	–1.87	(–7.47, 3.72)	0.507	–3.98	(–10.69, 2.73)	0.241

Results derived from multivariable linear regression models (total PQ score, total YSR T-score, and SOFAS score) and logistic regression models (PQ cut-off) examining the association between group status at recruitment (age 9–12 years) and clinical and functional outcomes at follow-up (age 17–21 years). Model 1, effect of group status adjusted for sociodemographic factors (sex, ethnicity, and caregiver occupation at wave 1); model 2, adjusted for sociodemographic factors and full-scale IQ measured at wave 1 (age 9-12 years); model 3, adjusted for sociodemographic factors and daily stressors measured at wave 2 (age 11–14 years). Bold font indicates statistically significant at 0.05 level.

*ASz* antecedents of schizophrenia, *FHx* family history of schizophrenia/schizoaffective disorder, *TD* typically developing, *ref* reference category, *PQ* prodromal questionnaire, *YSR* youth self-report, *SOFAS* social and occupational functioning assessment scale, *β* beta coefficient, *CI* confidence interval, *OR* odds ratio.

#### Sensitivity analyses

The primary models (model 1, adjusted for sex, ethnicity, and caregiver occupation) were repeated with the 4 individuals meeting both ASz and FHx criteria at wave 1 reassigned from the ASz to the FHx group. The pattern of results was largely unchanged (Supplementary Table S3) with the ASz group showing significantly higher total PQ scores and lower SOFAS scores compared to the TD group. However, the ASz group was no longer significantly more likely to meet PQ ‘probable prodrome’ criteria compared to the TD group, though the effect size was similar. Moreover, total YSR T-scores were not significantly higher in the ASz group relative to either the TD or FHx group in these sensitivity analyses.

## Discussion

The current study examined clinical and functional outcomes at age 17–21 years among youth recruited at age 9–12 years using a school-based screening procedure. Youth who presented a triad of putative ASz at recruitment were characterised by higher self-reported APS and overall psychopathology, and lower social and occupational functioning, relative to their typically-developing peers seven years later after adjusting for sex, ethnicity, and caregiver occupation at wave 1. These differences were largely unchanged after additionally adjusting for IQ at wave 1 but were substantially attenuated when daily stressor scores at wave 2 were included as a covariate. In contrast, the proportion of youth meeting CHR or FEP criteria at age 17–21 years was not markedly different across the ASz, FHx, and TD groups.

Our finding that ASz youth showed significantly higher total PQ scores and total YSR T-scores than the TD group and were more likely to obtain above-threshold PQ positive scores (≥8) at age 17–21 years is consistent with our earlier follow-up assessments (wave 2: 11–14 years) when ASz children had higher levels of internalising and externalising symptoms, social withdrawal, and PLEs than TD children^31,33^. These findings are perhaps unsurprising given that the presence of at least one PLE at age 9–12 years was an inclusion criterion for this group. In the Avon Longitudinal Study of Parents and Children cohort, which included assessments conducted at similar time-intervals to the present study, self-reported PLEs at age 12 were associated with a 2.4 fold increase in interview-assessed psychotic experiences at age 18 years^34^. We observed that differences between the ASz and TD groups on all clinical outcome measures were substantially attenuated after adjustment for wave 2 daily stressors score. These daily stressors included potentially discriminatory experiences (e.g. ‘other children make fun of me, tease me, or pick on me’) which may be particularly prevalent in the ASz and FHx groups, which include a larger proportion of individuals from ethnic minority backgrounds. Given that this measure captures both frequency of daily stressor exposure and distress associated with these events (which could be driven by underlying psychopathology), increased stress sensitivity may mediate the association between putative antecedents and later adverse clinical outcomes, making this a viable target for preventative efforts. Given the limited evidence showing that stress/trauma is associated with transition to psychosis in CHR samples^35^ (which may be due to the fact that these exposures are highly prevalent in CHR populations and have limited ability to predict transition over and above clinical measures) targeting stress sensitivity during the earlier risk phase, prior to the fulfilment of CHR criteria, may be advantageous.

The ASz group were also characterised by poorer social and occupational functioning at age 17–21 years compared to the TD group. However, it is important to note that the median score for the ASz group (73.0) corresponded to only ‘slight impairment’ and the vast majority (>90%) were in engaged full- or part-time education or employment. Importantly, the significant differences in SOFAS scores between ASz and TD groups were not explained by sex, ethnicity, or caregiver occupation, but were attenuated after adjusting for wave 1 IQ (an effect that was not observed in models examining APS and global psychopathology). Hence, the impairments in cognitive performance that we observed among ASz youth relative to their TD peers at earlier assessment phases (waves 1, 2, and 3)^28–30,36,37^ may partially contribute to poor long-term functioning in this group. Indeed, cognitive performance has been found to be associated cross-sectionally and longitudinally with social and role/occupational functioning among individuals with early psychosis (<5 years of illness duration)^38^ and individuals at CHR^39,40^. Such findings emphasise the importance of developing interventions targeting emerging cognitive impairments among youth with risk factors for psychosis.

In contrast to hypotheses, only three (9.1%) individuals in the ASz group fulfilled CHR/FEP criteria at the wave 4 follow-up, which was not markedly different to the proportion in the FHx (10.0%) and TD groups (7.5%), and not examined statistically due to small numbers. The prevalence of CAARMS CHR status that we observed across all three groups was higher than the 1.5% prevalence reported in the general population samples (mean age range 13.9–24.0 years)^41^. One potential explanation for this may be the young age of our sample, given the inverse association observed between age and CHR prevalence in clinical samples^41,42^. A further contributing factor may be that our cohort was recruited from inner-city London, a region which has been consistently shown to have the highest incidence of non-affective psychotic disorders (particularly schizophrenia) in England^43^. Factors hypothesised to contribute to the excess risk of psychotic disorders in adults residing in urban areas such as London (e.g. social deprivation, ethnic minority status, victimisation, cannabis use, air pollution, limited access to green spaces^44^) might plausibly increase the prevalence of CHR status in youth. The lack of difference that we observed in CHR prevalence across ASz and TD groups, in contrast to the PQ and SOFAS results, may reflect the fact that the latter were more likely to be female and CHR status is more common among female than male participants within general population samples^41^. In contrast, some studies conducted in non-clinical populations (including children, adolescents, and young adults), have reported that male participants show higher PQ scores than female participants^45,46^. It is plausible that these divergent findings across sex/gender groups are attributable to the different assessment methods, for example, girls/women may be more willing to disclose APS in interview than boys/men. However, in this small sample, we were unable to draw conclusions regarding the association between ASz status and later fulfilment of CHR criteria.

Our hypothesis that the FHx group would also be characterised by poorer clinical and functional outcomes relative to the TD group at follow-up was not supported. This finding is likely due to fact that the FHx group included both 1st and 2nd degree relatives, which enabled us to recruit a larger sample (our school-screening approach identified few individuals with a 1st degree relative) and examine the effect of high versus low familial loading for schizophrenia and related psychotic disorders. We have previously shown that FHx youth with higher familial loading (one 1st or ≥two 2nd degree relatives) are characterised by greater impairments in cognitive functioning (wave 1)^30,36^ and more pronounced hypothalamic-pituitary-adrenal (HPA) axis abnormalities (wave 2)^47^ than those with lower familial loading (one 2nd degree relative). Unfortunately, loss to follow-up meant that group sizes were too small to compare high and low familial loading groups on clinical and functional outcomes at wave 4. Although a recent study of at-risk youth aged 10–17 years observed that those with a 1st vs. 2nd degree affected relative did not differ on clinical or functional measures, all youth were help-seeking and were required to show functional impairment at intake^48^. Together, these findings suggest that, whilst studying individuals with a low familial loading for schizophrenia may help to delineate aetiological mechanisms, only those who experience functional impairments and help-seeking behaviour may truly be at elevated risk for poorer mental health outcomes. As such, the putative ASz approach represents a more viable option for selective prevention strategies than targeting a broadly-defined FHx group.

Collectively, the findings from our interim^28–31,33,36,37,49–54^ and present analyses, indicate that ASz status at age 9–12 years is associated with a divergent pattern of psychosocial, cognitive, and neurobiological development throughout childhood and adolescence and poorer clinical and functional outcomes in late adolescence/early adulthood. However, we have been unable to demonstrate (given our small sample) that ASz status confers increased risk for fulfilment of CHR criteria or transition to FEP. At the time our study commenced (nearly two decades ago), there was robust evidence supporting each of the individual putative antecedents that comprise the ASz triad, with prospective and follow-back studies showing these features to be more common among children who later developed schizophrenia and related psychotic disorders^22,55–64^. However, a recent umbrella review of risk and protective factors for psychotic disorders found only weak evidence (class IV) to suggest that PLEs, antisocial and externalising behaviour, and developmental delays (walking, standing, and sitting unsupported) were associated with later development of psychotic disorders^9^. Of note, this review did not examine internalising symptoms (due to a lack of previous reviews on this topic) or include an updated meta-analysis (published subsequently) examining PLEs and risk of psychotic disorders^23^. We originally reasoned that the presence of a triad (rather than a single) antecedent would be more strongly predictive of later psychosis; owing to our small sample size, and the absence of other similar cohorts, evidence to support this hypothesis is lacking.

In addition to the small sample, other limitations should be noted. First, as is the case in all high-risk studies, it is possible that the relatively poorer outcomes that we observed in the ASz group are driven by a ‘super healthy’ control group. However, the prevalence of CHR status in the TD group was higher than that reported in general population samples, suggesting that whilst these children were initially identified as being a ‘healthy’ control group, at least some developed clinically-relevant psychopathology by the wave 4 assessment. Second, whilst the determination of CHR/FEP status and SOFAS scores was performed blindly (using detailed interview transcripts), the researchers completing the CAARMS were not blind to risk status, potentially introducing interviewer bias. However, this would not have influenced self-reported PQ and YSR scores; moreover, the (relatively) high prevalence of CHR status in the TD group is at odds with this hypothesis. Finally, given the small size of the FHx group (even when including those who also met ASz criteria), statistical tests comparing this group to the ASz and TD groups were likely underpowered.

## Conclusion

Our 7-year follow-up of this unique cohort indicates that children identified from the general population as presenting a triad of putative ASz at age 9–12 years are characterised by higher levels of APS and global psychopathology and poorer social and occupational functioning relative to their typically-developing peers as they transition to adulthood. Whilst the small sample prevented us from examining the extent to which ASz status was associated with risk of fulfilling CHR criteria or transition to full-threshold psychotic disorder, our findings are consistent with our interim analyses showing that these youth are characterised by several features that are also observed among adults with schizophrenia and related psychotic disorders^16^. These features include social withdrawal^33^, poorer performance on measures of cognitive function, scholastic achievement, motor development, and facial emotional processing ability^28–30,37,51^, higher levels of involuntary movement abnormalities^54^, regional differences in grey and white matter volume^50^, abnormalities of brain function as indexed by event-related potentials^52,53^, and increased stress sensitivity^31^ relative to their TD peers. Whether these features indicate that children presenting ASz are at greater risk of developing psychosis or other psychiatric disorders at follow-up, however, is currently unknown. Larger, well-resourced studies are needed to determine whether community screening approaches can enable earlier detection of youth at elevated risk for psychosis.

## Methods

### Participant recruitment

Participants were drawn from the London Child Health and Development Study (CHADS), described in detail previously^16,18,19^. The present study includes individuals who fulfilled ASz, FHx, or TD criteria (defined below) at screening who were recruited to the ‘longitudinal selected sample’^16^ and invited to complete biennial, laboratory-based assessments throughout adolescence. The recruitment strategies for this cohort are described below; participation rates are provided in Supplementary Material.

The majority of participants were recruited using an epidemiologically-informed screening procedure, conducted in 73 primary schools in Greater London, UK^17^. Screening questionnaires were completed independently and anonymously in class by ~8000 children aged 9–12 years, with corresponding questionnaires (matched by code number) completed by caregivers at home. The questionnaires assessed a triad of well-replicated, putative ASz, defined as (i) a caregiver-reported delay in speech and/or motor development^18^, (ii) child-reported emotional symptoms and/or caregiver-reported peer relationship problems, conduct problems, and/or hyperactivity-inattention in the clinical range (approximately top 10th percentile based on UK norms) of the Strengths and Difficulties Questionnaire^65^, and (iii) the presence of at least one child-reported 'certainly-true' rating among nine PLEs measured with the Psychotic-Like Experiences Questionnaire for Children^17,18,66,67^. Caregiver questionnaires included additional items assessing family history of mental illness. We additionally screened the medical records of patients within the South London and Maudsley National Health Service (NHS) Foundation Trust to identify those with a diagnosis of schizophrenia or schizoaffective disorder who had a relative aged 9–12 years. These families were approached following liaison with the patient’s care worker.

Children presenting all three putative antecedents were eligible for the ASz group. Children with a 1st or 2nd degree relative with schizophrenia/schizoaffective disorder (as identified in screening questionnaires or via medical records, and subsequently confirmed by caregiver interview using the Family Interview for Genetic Studies: FIGS^68^), were eligible for the FHx group. In the present study, children who met both ASz and FHx group criteria were included in the ASz group. TD children presented none of the putative antecedents and had no FHx, schizoaffective disorder, or bipolar disorder as confirmed using the FIGS. Exclusion criteria for all groups were (i) insufficient English language ability (child or caregiver) to complete assessments, (ii) the presence of a neurological condition that affected milestone attainment or current functioning (e.g. epilepsy or cerebral palsy), (iii) a diagnosis of autism, Asperger’s or diagnosed learning disability (IQ < 70), or (iv) a prior psychotic episode or use of antipsychotic medication.

### Procedure

Participants completed a baseline assessment at age 9–12 years (wave 1), and three follow-up assessments throughout childhood/adolescence (wave 2: ages 11–14; wave 3: 13–16; and wave 4: 17–21 years), comprising a range of biological, psychosocial, and cognitive measures. The current study examines clinical and functional outcome measures obtained at wave 4. Clinical interviews were conducted by two of the authors (AEC and RER) who, in addition to completing training on the assessment measures, received guidance and supervision from an experienced mental health practitioner from a specialist CHR service. Outcome assessments were scored by a single rater (AEC), blinded to risk group, from video recordings obtained at the session.

Children and their caregivers provided written informed assent and consent, respectively, to participate in waves 1–3 assessments; most participants attended the wave 4 assessment alone and provided written consent to participate. Ethical approvals were granted by the Joint South London and Maudsley and the Institute of Psychiatry NHS Research Ethics Committee and the King’s College London, Psychiatry, Nursing and Midwifery Research Ethics Committee.

### Measures

#### Sociodemographic characteristics

At wave 1, caregivers provided information on participant age, biological sex, and ethnicity (the latter determined using information from the FIGS interview and subsequently categorised as white, black, and other) and caregiver occupation (coded as managerial/professional, intermediate, and routine and manual, according to the UK National Statistics Socio-economic Classification^69^). Caregiver occupation data were missing for 11 participants at the initial assessment but were collected at the wave 2 or 3 assessment for 6 and 4 of these participants, respectively.

#### Clinical and functional outcomes at age 17–21 years

Participants completed the PQ^70^ at wave 4, a widely-used screening tool for the CHR state^71^. This self-report measure includes 92 items (rated ‘true’/ ‘false’) assessing positive (45 items), negative (19 items), disorganized (13 items), and general (15 items) symptoms, which were summed to create a total PQ score. Scores of ≥8 and ≥18 on the positive symptom scale are validated thresholds for identifying possible CHR cases in highly enriched samples and general mental health help-seeking populations, respectively^72^. For our community sample, we report descriptive statistics for both cut-off scores, but apply the threshold of ≥8 for statistical analyses.

Global psychopathology was assessed using the YSR, an extensively-used measure which exhibits high reliability and validity^73^. This 112-item questionnaire captures problems occurring during the past six months on a three-point scale (0 ‘not true’, 1 ‘somewhat true or sometimes true’, or 2 ‘very true or often true’). In the present study, we derived total YSR scores (sum of scores from 8 scales: anxious/depressed; withdrawn/depressed; somatic complaints; social problems; thought problems attention problems; rule-breaking behaviour; aggressive behaviour) which were converted to age- and sex-normed T-scores.

Participants provided detailed information regarding current education and employment status as part of the wave 4 clinical interview. This included details of educational and vocational qualifications, attendance, and performance at school/college/university/work. Participants were also asked to rate their levels of satisfaction with their current education/employment situation on an 11-point scale (0 ‘not at all’ to 10 ‘extremely’) and indicate whether this had changed during the past year. These data were used to determine scores on the SOFAS^74^, a widely-used measure of global functioning that yields a score from 1 to 100, with higher scores indicating better functioning. The SOFAS was chosen as a measure of global functioning as scores on this scale are used to determine CHR status (detailed below).

At wave 4, the full CAARMS^75^ was administered to determine CHR status and onset of FEP. The CAARMS is a semi-structured assessment tool that assesses psychopathology across seven domains: positive symptoms, cognitive change, emotional disturbance, negative symptoms, behavioural change, motor/physical changes, and general psychopathology. Intensity and frequency/duration ratings (each scored on scales ranging 0–6) from the four positive symptoms (unusual thought content, non-bizarre ideas, perceptual abnormalities, and disorganised speech) are used to determine eligibility for three CHR subgroups: (i) APS (positive symptoms of subthreshold intensity or frequency), (ii) brief, limited, intermittent psychotic symptoms (BLIPS, frank psychotic symptoms of less than one week duration that spontaneously resolve), and (iii) trait vulnerability (schizotypal personality disorder or a 1st degree relative with psychotic disorder). All three groups are required to show a 30% reduction in SOFAS scores within the past 12 months, or a SOFAS score of ≤50 for 12 months or longer. FEP threshold is reached when one or more of the four positive symptoms are of sufficient severity and frequency^75^.

#### Covariates

General intelligence (full-scale IQ) was assessed at wave 1 from two subtests (vocabulary and matrix reasoning) of the Wechsler Abbreviated Scale of Intelligence^76^, which is suitable for use with both children and adults, affording longitudinal assessment^26^. Five participants did not complete IQ testing at wave 1, so IQ scores from wave 2 (*n* = 3) and wave 3 (*n* = 2) assessments were used for these participants. Psychosocial stress was assessed at wave 2 using a 37-item self-report measure^77^ which captured frequency of exposure (range: 0 ‘never’ to 3 ‘often’) to daily stressors pertaining to school-work, peers, teachers, and home during the past six months, and the degree of distress (range: 0 ‘not at all’ to 3 ‘a lot’) experienced in relation to each event. The daily stressor scale was chosen as a covariate for the present study based on our previous work showing that daily stressor scores distinguished between ASz and TD groups^31^ and, in a larger, partially overlapping sample, were independently associated with total PQ scores at wave 4 after adjusting for prior psychopathology^32^. As previously^32^, ratings across each item were summed to derive a total exposure score and a total distress score, with principal component analysis applied (scores on the first component retained) to derive an overall daily stressor score.

### Statistical analysis

Analyses were performed using Stata Version 16^78^. Participants who completed the wave 4 assessment were compared to those lost to follow-up on risk group status, sociodemographic variables, full-scale IQ, and daily stressor score, using Chi-squared and Fisher’s exact tests for categorical variables and Mann–Whitney U tests for continuous variables (due to unequal group sizes and departures from normality). Chi-squared/Fisher’s exact tests and Kruskal–Wallis tests were then used to compare ASz, FHx, and TD groups on sociodemographic variables. To identify covariates for inclusion in multivariable analyses, we examined whether sociodemographic variables, full-scale IQ, and daily stressor scores were associated with outcome variables using Pearson/Spearman’s correlations (for continuous-continuous pairings), *t*-tests/Mann–Whitney tests/Kruskal–Wallis tests (for categorical-continuous pairings) and Chi-squared/Fisher’s exact tests (for categorical-categorical pairings).

Multivariable regression analyses were conducted to examine the effect of risk group at wave 1 on outcome variables at wave 4; linear and logistic regression models were used for continuous and binary outcomes, respectively. Following each model, pairwise comparisons of marginal linear predictions were performed (‘pwcompare’, with the default ‘noadjust’ option) for risk group status, providing a comparison of each risk group with every other risk group. We first performed models that were adjusted for sociodemographic factors that were found in preliminary analyses to be associated with both group status and outcome variables at the *P* < 0.1 level (model 1). In models 2 and 3, we included full-scale IQ and daily stressor score, respectively, as additional covariates. Checks of multicollinearity between all predictor variables using the ‘collin’ command confirmed that this assumption was not violated. For linear regression models, we additionally checked that the residuals were approximately normally distributed using kernel density plots. Lastly, for all outcomes, we performed sensitivity analyses in which individuals who fulfilled both ASz and FHx criteria at screening were reassigned to the FHx group from the ASz group. Statistical significance was set at *P* < 0.05 for all regression models.

## Supplementary information

Supplemental methods

Supplemental tables

## Data Availability

This study reports on data collected as part of the “London Child Health and Development Study”. These data cannot be made publicly available due to ethical restrictions, with participant consent permitting only research team members to access the data provided. Anonymised data from the studies are held by the Principal Investigator Prof. Kristin R. Laurens (Kristin.Laurens@qut.edu.au). Those interested in obtaining these data should contact Prof. Laurens to request appropriate approval for access.
