# Supplementary material for: Clinical and functional outcomes at 7-year follow-up of children presenting putative antecedents of schizophrenia at age 9-12 years
Source: Schizophrenia (Heidelb). 2024 Sep 30;10(1):83. doi: 10.1038/s41537-024-00507-8 (PMC11442655; doi:10.1038/s41537-024-00507-8)
Supplement: Supplementary file 1 — Supplemental methods [file 41537_2024_507_MOESM1_ESM.docx]

**SUPPLEMENTARY METHODS**

**Clinical and functional outcomes at 7-year follow-up of children presenting putative antecedents of schizophrenia at age 9-12 years**

**Cullen AE, Roberts RE, Fisher HL, Laurens KR**

**PARTICIPATION RATES**

In total, 7966 children aged 9-12 years (representing 95% of eligible children enrolled at participating schools) completed screening questionnaires at school (4% of parents and 1% of children refused the child’s participation, for reasons unknown). Subsequently, 1504 caregivers (18.9%) completed corresponding questionnaires and returned these via reply-paid mail. Among these, 799 families provided contact information (names and addresses) and indicated willingness to be contacted to participate in further research.

From medical record screening, we identified 36 patients with schizophrenia or schizoaffective disorder who had a relative aged 9-12 years. Contact information for the child relative and their primary caregiver were obtained via liaison with the patient’s care worker.

Of the 835 families (799 + 36) who agreed to be contacted for research, we invited 240 to participate in research assessments at the Institute of Psychiatry, Psychology & Neuroscience, King’s College London, UK. These 240 cases were selected because they presented with either:

(a) None of the three factors defining the antecedent triad and no family history of severe mental illness (recruited to the typically-developing group [TD]).

(b) All three of the antecedents defining the triad (recruited to the ASz group).

(c) A family history of schizophrenia/schizoaffective disorder, indicated on caregiver screening questionnaire or in clinical records and subsequently confirmed using the Family Interview for Genetic Studies (FIGS) ^1^ (recruited to the FHx group).

(d) Two of the three factors within the antecedent triad (initially recruited to form an alternative comparison group).

Additional cases within the 835 families who met eligibility criteria could not be approached due to limited study resources. Other families could not be reached as their contact details had changed subsequent to screening.

Among the 240 families invited to attend the first assessment session (wave 1; age 9-12 years), 150 participated. Of these, 112 met group criteria and were actively pursued at subsequent follow-up assessments, including: 45 TD, 35 ASz, 26 FHx, and 6 children meeting both ASz and FHx criteria. The remaining 38 children were those who either presented with 2 of the 3 antecedents at screening (only a subset of whom were followed-up due to limited resources), or those who were not invited to participate in subsequent assessments because a neurological abnormality was identified in an MRI scan or the child had received treatment for a psychiatric disorder at the time of initial assessment.

Comparisons between the 93 (of 112) participants who completed the wave 4 assessment at age 17-21 years and those who did not are presented in Supplementary Table S1.
