# Supplementary material for: Clinical and functional outcomes at 7-year follow-up of children presenting putative antecedents of schizophrenia at age 9-12 years
Source: Schizophrenia (Heidelb). 2024 Sep 30;10(1):83. doi: 10.1038/s41537-024-00507-8 (PMC11442655; doi:10.1038/s41537-024-00507-8)
Supplement: Supplementary file 2 — Supplemental tables [file 41537_2024_507_MOESM2_ESM.docx]

**SUPPLEMENTARY TABLES**

**Clinical and functional outcomes at 7-year follow-up of children presenting putative antecedents of schizophrenia at age 9-12 years**

**Cullen AE, Roberts RE, Fisher HL, Laurens KR**

[Table S1. Comparison of participants who completed the wave 4 assessment and those lost to follow-up 2](#_Toc175575146)

[Table S2. Associations of sociodemographic characteristics, full-scale IQ at wave 1, and daily stressors at wave 2 with clinical and functional outcomes at wave 4 in the total sample (N=93) 3](#_Toc175575147)

[Table S3. Sensitivity analyses examining the association between risk status at recruitment and clinical and functional outcomes at 7-year follow-up, with the four individuals meeting both antecedent and family history criteria assigned to the family history group 4](#_Toc175575148)

Table S1. Comparison of participants who completed the wave 4 assessment and those lost to follow-up

|  |  | **Total sample recruited at baseline (N=112)** | | | | |
| --- | --- | --- | --- | --- | --- | --- |
| **Characteristics** | **Wave** | **Assessed at follow-up (n=93)** | | **Lost to follow-up**  **(n=19)** | | **Statistical test** |
| Group status at recruitment | **SC** |  |  |  |  |  |
| ASz |  | 33 | (35.5) | 8 | (42.1) | *FE*, *P*=0.382 |
| FHx |  | 20 | (21.5) | 6 | (31.6) |  |
| TD |  | 40 | (43.0) | 5 | (26.3) |  |
| Age at screening (years), Med. (IQR) | SC | 10.33 | (1.20) | 10.35 | (1.53) | *MW*=0.74, *P* =0.459 |
| Sex (male), n (%) | 1 | 45 | (48.4) | 13 | (68.4) | χ^2^=2.54, *P* =0.111 |
| Ethnicity, n (%) | 1 |  |  |  |  |  |
| White |  | 49 | (47.3) | 8 | (9.7) | *FE*, *P* =0.601 |
| Black |  | 24 | (25.7) | 7 | (5.3) |  |
| Other |  | 20 | (19.9) | 4 | (4.1) |  |
| Caregiver occupation, n (%) | 1^a^ |  |  |  |  |  |
| Managerial/professional |  | 63 | (68.5) | 6 | (33.3) | *FE*, *P* =0.009 |
| Intermediate |  | 17 | (18.5) | 5 | (27.8) |  |
| Routine and manual |  | 12 | (13.0) | 7 | (38.9) |  |
| Full-scale IQ, mean (SD) | 1^b^ | 107.75 | (14.59) | 94.63 | (15.72) | *MW*=-2.89, *P*=0.004 |
| Daily stressors PC score, mean (SD) | 2 | 0.05 | (1.26) | -0.26 | (1.84) | *MW*=-0.54, *P* =0.593 |

IQR, interquartile range; ASz, antecedents of schizophrenia; FHx, family history of schizophrenia/schizoaffective disorder; TD, typically developing. *MW*, Mann-Whitney U test; χ^2^, Pearson chi-squared; *FE*, Fisher’s exact test; PC, principal component score. ^a^ Collected at wave 2 (n=6) or 3 (n=4) due to missing data at wave 1; ^b^ collected at wave 2 (n=3) and wave 3 (n=2) due to missing data at wave 1. Missing data, caregiver occupation (n=2), daily stressor PC score (n=6).

Table S2. Associations of sociodemographic characteristics, full-scale IQ at wave 1, and daily stressors at wave 2 with clinical and functional outcomes at wave 4 in the total sample (N=93)

|  | **Clinical and functional outcomes assessed at wave 4** | | | |
| --- | --- | --- | --- | --- |
| **Measure (assessment wave)** | **PQ total score** | **PQ cut-off**  **(≥8 positive scale)** | **Total YSR T-score** | **SOFAS score** |
| Age at screening (SC) | *r_s_* = 0.024, *P* = 0.822 | *MW* = -0.558, *P* = 0.577 | *r_s_* = -0.010, *P* = 0.927 | *r_s_* = 0.095, *P* = 0.366 |
| Age at follow-up (wave 4) | *r_s_* = -0.015, *P* = 0.888 | *MW* = 1.269, *P* = 0.205 | *r_s_* = -0.120, *P* = 0.273 | *r_s_* = -0.121, *P* = 0.247 |
| Time lapse | *r_s_* = -0.052, *P* = 0.618 | *MW* = 1.124, *P* = 0.261 | *r* = -0.004, *P* = 0.966 | *r_s_* = -0.089, *P* = 0.397 |
| Sex (wave 1) | *MW* = 1.958, *P* = 0.050 | *χ^2^* = 2.935, *P* = 0.087 | *t* = 1.421, *P* = 0.159 | *MW* = -0.517, *P* = 0.605 |
| Ethnicity (wave 1) | *KW* = 6.323, *P* = 0.043 | *χ^2^* = 4.904, *P* = 0.086 | *KW* = 1.929, *P* = 0.381 | *KW* = 7.329, *P* = 0.026 |
| Caregiver occupation (wave 1) | *KW* = 7.000, *P* = 0.030 | *FE*, *P* =0.256 | *KW* = 1.811, *P* = 0.404 | *KW* = 14.898, *P* < 0.001 |
| Full-scale IQ (wave 1) | *r_s_* = -0.173, *P* = 0.098 | *MW* = 0.606, *P* = 0.544 | *r* = 0.001, *P* = 0.992 | *r_s_* = 0.456, *P* < 0.001 |
| Daily stressors PC score (wave 2) | *r_s_* = 0.412, *P* < 0.001 | *MW* = -2.534, *P* = 0.011 | *r* = 0.425, *P* < 0.001 | *r_s_* = -0.362, *P* < 0.001 |

PQ, Prodromal Questionnaire; YSR, Youth self-report; SOFAS, Social and Occupational Functioning Assessment Scale; *r,* Pearson’s correlation coefficient; *r_s_*, Spearman’s rho; *MW,* Mann-Whitney U test; *KW*, Kruskal Wallis test; *FE*, Fisher’s exact test.

Table S3. Sensitivity analyses examining the association between risk status at recruitment and clinical and functional outcomes at 7-year follow-up, with the four individuals meeting both antecedent and family history criteria assigned to the family history group

|  | **PQ total score** | | | **PQ cut-off**  **(≥8 positive scale)** | | | **Total YSR T-score** | | | **SOFAS score** | | |
| --- | --- | --- | --- | --- | --- | --- | --- | --- | --- | --- | --- | --- |
|  | ***β*** | **(95% CI)** | ***P*** | **OR** | **(95% CI)** | ***P*** | ***β*** | **(95% CI)** | ***P*** | ***β*** | **(95% CI)** | ***P*** |
| Pairwise comparisons |  |  |  |  |  |  |  |  |  |  |  |  |
| ASz vs. TD (ref) | **8.91** | **(1.77, 16.05)** | **0.015** | 3.01 | (0.80, 11.26) | 0.102 | **4.67** | **(-0.69, 10.04)** | **0.087** | **-8.45** | **(-14.35, -2.55)** | **0.006** |
| ASz vs. FHx (ref) | 1.90 | (-6.15, 9.95) | 0.640 | 0.86 | (0.22, 3.39) | 0.824 | 3.67 | (-2.45, 9.80) | 0.236 | -2.52 | (-9.17, 4.13) | 0.453 |
| FHx vs. TD (ref) | 7.01 | (-0.69, 14.70) | 0.074 | 3.52 | (0.89, 13.89) | 0.073 | 1.00 | (-4.84, 6.84) | 0.735 | -5.93 | (-12.29, 0.42) | 0.067 |

Results derived from multivariable linear regression models (total PQ score and SOFAS score) and logistic regression models (PQ cut-off) examining the association between risk status at recruitment (age 9-12 years) and clinical and functional outcomes at follow-up (age 17-21 years) adjusted for sex, ethnicity, and caregiver occupation at baseline. ASz, antecedents of schizophrenia; FHx, family history of schizophrenia/schizoaffective disorder; TD, typically developing; ref, reference category; PQ, Prodromal Questionnaire; SOFAS, Social and Occupational Functioning Assessment Scale; *β*, beta coefficient; CI, confidence interval; OR, odds ratio. Bold font indicates statistically significant at 0.05 level.
